# Statistical analyses on effective removal of cadmium and hexavalent chromium ions by multiwall carbon nanotubes (MWCNTs)

**DOI:** 10.1016/j.heliyon.2020.e04174

**Published:** 2020-06-08

**Authors:** K.S. Obayomi, J.O. Bello, M.D. Yahya, E. Chukwunedum, J.B. Adeoye

**Affiliations:** a Department of Chemical Engineering, Landmark University Omu-Aran Kwara State, Nigeria; b Department of Chemical Engineering, Federal University of Technology Minna Niger State, Nigeria

**Keywords:** Chemical engineering, Castor seed, Activated carbon, Transition metals, MWCNTs, Adsorption isotherm

## Abstract

In this work, multiwall carbon nanotubes (MWCNTs) developed from cobalt-ferrite catalyst on activated carbon (from castor seed), was used as an adsorbent for the removal of cadmium and hexavalent chromium ions. The effectiveness of the adsorbent for the uptake of Cd(II) and Cr(VI)ions from aqueous solution was investigated in a process batch adsorption study. The developed activated carbon and MWCNTs were characterized by Brunauer-Emmett-Teller (BET) surface area analysis, Fourier Infrared Spectroscopy (FT-IR) and Scanning Electron Microscopy (SEM) for the determination of surface area, functional group, and surface morphology, respectively. The BET surface area of activated carbon and developed adsorbent from Co–Fe/AC was 230.24 and 372.42 m^2^/g, respectively. The operational parameters evaluated on the adsorption efficiency were solution pH, temperature, adsorbent dosage initial metal ions concentration, and contact time. The adsorption of Cd(II) and Cr(VI) were found to have attained equilibrium positions in 60 min for the concentration range tested, respectively. The four linearized adsorption isotherm models; Langmuir, Freundlich, Temkin and Dubinin Radushkevich (D-R) tested, when compared, revealed that Langmuir isotherm fitted well to the experimental data judging from the higher correlation coefficient values (R^2^) and lower values of the error functions (chi-square (χ^2^), the sum of square error (ERRSQ/SSE) and the sum of absolute error (EABS))with monolayer adsorption capacities of 404.858 and 243.902 mg/g for Cd(II) and Cr(VI) ions, respectively. Adsorption kinetic models investigated by pseudo-first-order, pseudo-second-order, Elovich, and intraparticle diffusion showed the conformity of pseudo-second-order model to the process adsorption as informed by the higher values R^2^ and Adj, R^2^, maximum log-likelihood and smaller ERRSQ/SSE, χ^2^, Akaike information criterion (AIC), Bayesian information criterion (BIC), and Hannan-Quinn information criterion (HQIC). The intraparticle diffusion model plots indicated that intraparticle diffusion was not the only rate-limiting step. Thermodynamic adsorption parameters (ΔH^o^ and ΔG^o^, ΔS^o^) showed that the adsorption of Cd (II) and Cr (VI) ions was spontaneous, endothermic, and increased in randomness between the adsorbate-adsorbent. The mean adsorption energy (E), the heat of adsorption (ΔH^o^), and activation energy (E_a_) values, revealed the adsorption mechanism of Cd(II) and Cr(VI) onto MWCNTs as a combination of chemical and physical adsorption but dominated more by chemical adsorption.

## 1 Introduction

Water is particularly important to the existence of living organisms in the world. However, human activities which include population growth, industrialization increase, and urbanization have caused rapid contamination, compromising the availability of potable water [1]. The release of harmful substances like heavy metals, due to human actions, has posed environmental problems, affecting human health [2, 3]. Heavy metals like chromium and cadmium when exceeding their tolerance level in water are extremely toxic. Chromium and cadmium showed toxic biological effects such as damage to bones and kidney, death in humans, acute effects in children, necrosis nephritis, bronchitis, abdominal pain, liver damage, skin irritation, respiratory cancer, and irritation of gastrointestinal mucosa [4]. Chromium exists as two stable species; Cr (III) which is a macronutrient, and the toxic hexavalent Cr (VI) [5]. Recently, rapid development globally has led to the discharge of these heavy metals into water bodies, which is dangerous to humans and their environment. Thus, it is imperative to reduce these heavy metals or remove them completely using efficient and suitable technologies to prevent hazardous effects on the environment and human health. The reason being that they exhibit non-biodegradable and bioaccumulation tendencies in plants and animals [6, 7].

The decontamination of heavy metals from wastewater before their discharge into the landmass, channels, rivers, and ocean using various technologies like adsorption, reverse osmosis mechanism, coagulation, filtration, electrochemical treatment, and sedimentation process, has gained attention over the years [8, 9]. Adsorption process, amongst other techniques, is considered the best because of its advantages including, adsorbent regeneration, efficiency and effectiveness, simplicity, good adsorption capacity, and economical and eco-friendly use in heavy metals removal from contaminated solutions [10, 11]. The use of an effective and efficient adsorbent in the uptake of heavy metal from wastewater using adsorption techniques is of utmost significance. However, the use of nanomaterials (CNTs) with excellent mechanical and chemical properties, thermal stability, high surface area, and high adsorption capacity, has been given much credit [12, 13].

Several natural and synthetic materials have served as an adsorbent for the removal of heavy metals and these include zeolites, silica gel, clays, and chitosan. However, in recent times, the application of nanotechnology for wastewater treatment has gained worldwide attention [14, 15, 16]. This emerging research of Carbon nanotubes as an adsorbent for wastewater decontamination is attributable to its structural uniqueness comprising high porosity, hollow structure, and a large area of reaction [17]. The outer surface of individual nanotubes, the surface exterior where two adjacent parallel tubes meet, the groove which exists on the nanotube periphery bundles, the interstitial spaces between individual nanotubes in the bundles, and the cylindrical interior of individual nanotubes, are the numerous pores and sites available for the use of CNTs as an adsorbent for adsorption technique [18]. CNTs, alongside other adsorbents when compared, have a relatively larger surface area, high reactivity, photocatalytic activity, extraordinary surface morphology, an enormous number of active sites, and strong chemical and mechanical properties for its use as an adsorbent [19].

In this work, MWCNTs adsorbent was developed in a catalytic vapour deposition (CVD) reactor using castor seed activated carbon as catalyst support and was further used for Cr(VI) and Cd(II) ions uptake from aqueous solution. Equilibrium adsorption, thermodynamic, and kinetic studies were used to evaluate the effective performance of Cr(VI) and Cd(II) ions removal on the adsorbent active sites surface.

In summary, to the best of our knowledge, there has not been any studies in the literature regarding the use of activated carbon prepared from castor seed as catalyst support on Cobalt-Ferrite for the development of MWCNTS, as an adsorbent for excellent Cd(II) and Cr(VI) uptake. Therefore, there is an essential need to achieve a better percentage of uptakes with minimal cost. Also, error functions and information criterions analysis were incorporated together with the correlation coefficient, in measuring the suitability of the model fitness to the adsorption equilibrium and kinetic data.

## 2 Materials and method

### 2.1 Materials

Chemicals which include, potassium dichromate (K_2_Cr_2_O_7_), Cadmium nitrate (Cd(NO_3_)_2_.4H_2_0) Cobalt nitrate hexahydrate (Co(NO_3_)_2_·6H_2_O), Iron nitrate nonahydrate (Fe(NO_3_)_3_·9H2O), sodium hydroxide (NaOH), potassium hydroxide (KOH), and concentrated hydrochloric acid (HCl) of analytical grade, were supplied by Sigma- Aldrich with 98–99.99 % purity. Gases such as Acetylene (C_2_H_2_) and argon (Ar) of 99.99% purity were purchased at British Oxygen Company (BOC Gases Plc, Lagos, Nigeria). Castor seed was obtained from the farmland in Landmark University, Omu-Aran Kwara State, Nigeria.

### 2.2 Activated carbon development

The castor seeds were washed and dried in an oven overnight at 108 °C to attain a constant mass. The dried castor seeds were weighed into a crucible and loaded into a heating furnace set at 350 °C for 60 min, 5 °C/min heating rate, and 98 % purified nitrogen was flown through the furnace at 100 cm^3^/min. The operating condition (time and temperature) was obtained as a result of initial optimization studies from the literature. The char was cooled to room temperature and reduce to a particle size of 125 μm. The char prepared was impregnated with KOH at a ratio of 2:1 g/g (KOH: char). The impregnated char was left overnight, and after which it was thoroughly washed with distillate water to attain a neutral pH 7. The prepared sample was kept in the oven for 6 h at 108 °C to reduce the moisture content. The sample was removed after the time elapsed and was stored for further use in an airtight bottle.

### 2.3 Synthesis of activated carbon support cobalt-ferrite catalyst

Bi-metallic catalyst (Cobalt-ferrite), which was supported on activated carbon prepared from the castor seed, was developed using the wet impregnation method. 0.5 M each of Co(NO_3_)_2_.6H_2_O and Fe(NO_3_)_3_.9H_2_O was measured and transferred into 250 mL Erlenmeyer flasks with 100 mL of distillate water at room temperature for dissolution. Preparing the salt solution, 10 g of the prepared activated carbon was impregnated, and the mixture was vigorously stirred on a magnetic stirrer to remove moisture and form a semi-dried cake for 1 h at 110 °C and at a stirring speed of 240 rpm. The mixtures after stirring were left overnight to soak, after which the slurries were oven-dried for 6 h at 120 °C. The dried sample was further calcined at 400 °C for 1 h under the flow of argon gas; allowed to cool at room temperature, grounded with mortar and pestle, and reduced to the particle size of 125 μm. The Co–Fe/AC catalyst was then stored for use in CNTs production.

### 2.4 CNTs preparation

In the preparation of CNTs, the described procedure by Karim *et al.* [20], in a CVD reactor was employed. 2 g of the prepared Co–Fe/AC catalyst was weighed and channelled into the horizontal reactor's tube via the quartz tube. Air and other gaseous impurities from the quartz tube reactor were purged at 20 mL/min, from room temperature to the reaction temperature of 700 °C. The acetylene gas and reactant were allowed to pass through the catalytic reactor for 30 min at 100 mL/min, while the inert gas flow which serves as the carrier gas was increased to 200 mL/min. The acetylene flow was cut off and the reactor was purged at 20 mL/min until it was cooled to room temperature. The developed adsorbent was then removed, stored in an airtight container, and analysed to determine its surface properties.

### 2.5 Characterization of prepared activated carbon and CNTs adsorbent

The developed activated carbon, cobalt-ferrite/activated carbon catalyst, and CNTs adsorbent were characterized using Fourier Transformed Infrared Spectroscopy (FT-IR) to determine the functional group; Brunauer Emmett Teller (BET) to determine the surface area; and Scanning Electron Microscope (SEM) for the surface morphology, respectively. BET surface area was performed using BET (Micrometrics ASAP 2020), USA. Surface morphology was determined using the microanalysis scheme Oxford INCA/ENERGY-350, UK. While, FTIR spectra were registered by Perkin-Elmer infrared spectrophotometer, USA.

### 2.6 Experimental batch adsorption study

Equilibrium batch adsorption studies were tested using distilled water used as a solvent with Cd (II) and Cr(IV). 1000 mg/L stock solutions of Cd(II) and Cr(VI) ions were prepared by known weight each of Cd(NO_3_)_2_.4H_2_0 and K_2_Cr_2_O_7_dissolution in distilled water (1000 mL) and was used further to prepare the adsorbates (Cd(II) and Cr(IV)) solution of required concentrations (50, 100 and 150 mg/L) in a separate flask. In this experimental batch adsorption study, the experimental procedure described by [21, 22] was adopted. Equilibrium batch adsorption studies with various concentrations (50, 100 and 150 mg/L) of Cr(VI) and Cd(II)at different temperatures (35, 45 and 55 °C) in a set of Erlenmeyer flasks of 250 mL were carried out. To a set of 250 mL Erlenmeyer flasks, 100 mL of initial adsorbates of Cd(II) and Cr(VI) concentration of 50–150 mg/L range were introduced and 0.1 g of the prepared MWCNTs adsorbent was added. The pH solution of 2 and 8 for Cr(VI) and Cd(II) was maintained throughout the adsorption process. The flasks containing adsorbates of various concentrations were positioned on a magnetic shaker attuned at 35 °C for a shaker speed of 240 rpm for 2 h. Thereafter, Cd(II) and Cr(VI) ions samples were taken at different concentrations, filtered through a 45 mm micropore-filter membrane. Cd (II) ions samples were analysed using Atomic Absorption Spectroscopy (Perkin Elmer, Model A Analyst 200), while Cr(VI) ions samples were determined with the aid of an ultraviolet-visible spectrophotometer (Cary60, Agilent, USA), employing the 1,5-diphenylcarbazide Spectrophotometric method at 540nm. The entire adsorption experimental process was repeated for both Cr(VI) and Cd(II) ions with another set of flasks when the magnetic stirrer was attuned to 45 and 55 °C, respectively. The amount adsorbed of Cr(VI) and Cd(II) ions in mg/g at equilibrium was evaluated using Eq. (1)

(1) $$\text{qe}=\frac{\left(C_{O}-C_{e}\right)V}{W}$$

Where q_e_ is the metals ions adsorbed amount at equilibrium in mg/g; C_o_ and C_e_ are the initial and equilibrium metal ions concentrations in mg/L; V is the adsorbate volume in L, and W is the adsorbent weight in g.

Adsorption kinetics studies of Cr(VI) and Cd(II) ions adsorbed onto the prepared adsorbents were investigated at a fixed interval of time. The adsorbed amount of metal ions at a time it was determined using Eq. (2)

(2) $$\text{qt}=\frac{\left(C_{0}-C_{t}\right)V}{W}$$

Where C_t_ is the concentration of metal ions at any time (mg/L) and q_t_ is the metals ions adsorbed amount at different time interval in mg/g.

## 3 Results and discussion

### 3.1 Characterization

**Figure 1:**
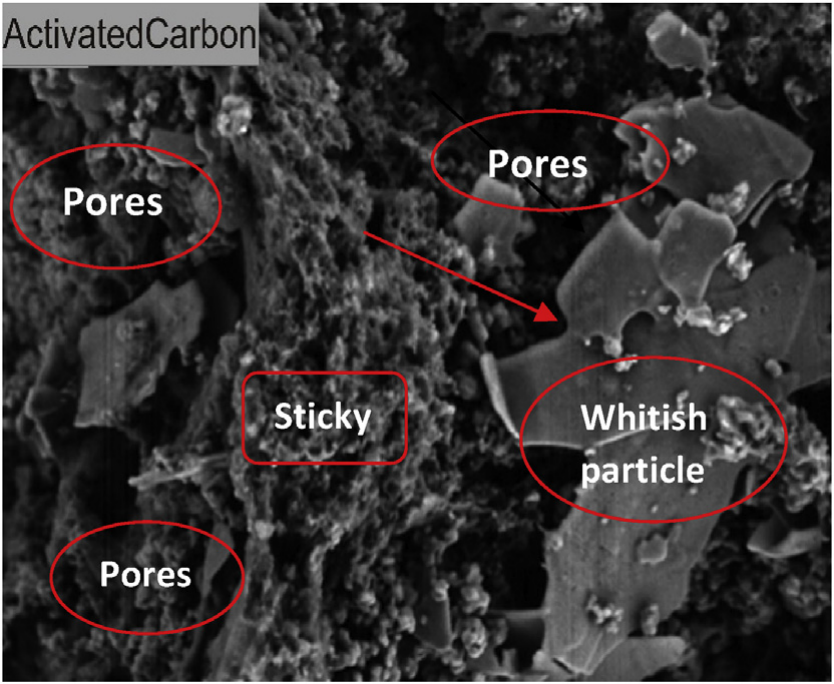
SEM image of activated carbon prepared from castor seed.

**Figure 2:**
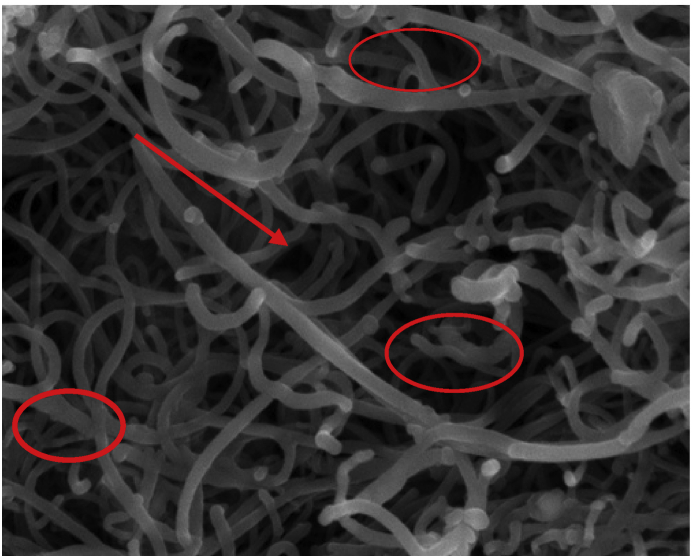
SEM image of CNTs prepared from Co–Fe catalyst supported on activated carbon.

Surface morphology of the developed activated carbon and CNTs as depicted in Figures 1 and 2, were discovered using SEM techniques. The developed activated carbon surface as seen in Figure 1 shows a sticky structure and a whitish particle on the surface as a result of incomplete combustion during the carbonization reaction. The hollow structure on the developed activated carbon, which resulted in the porous surface, may be attributed to the heat treatment with a suitable activating agent, resulting in pores development. The SEM image of the prepared CNTs from transition metal (Co–Fe) supported on activated carbon was presented in Figure 2. The result shows a web-like with irregular tubes having tiny particles with little agglomeration on the surface. The carbon conversion enhancement in the nucleation process of the CNTs formation in a catalytic vapour deposition (CVD) reactor may be due to the incorporation of Co–Fe catalyst supported on activated carbon and produced from the castor seeds. It can be observed from the CNTs SEM image that activated carbon offers excellent support on the transition metals. The adsorbent active centres are considered as the receiver of the adsorbate during the adsorption process.

**Figure 3:**
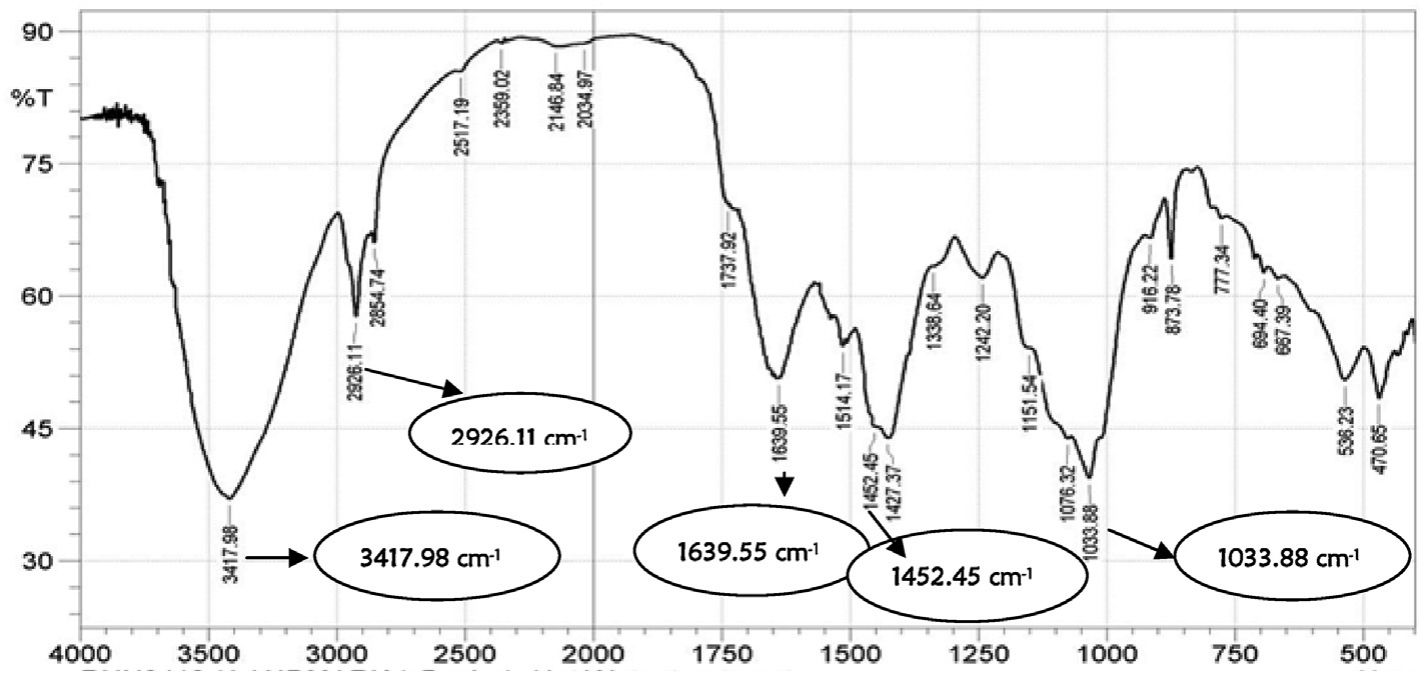
FT-IR of castor seed activated carbon.

**Figure 4:**
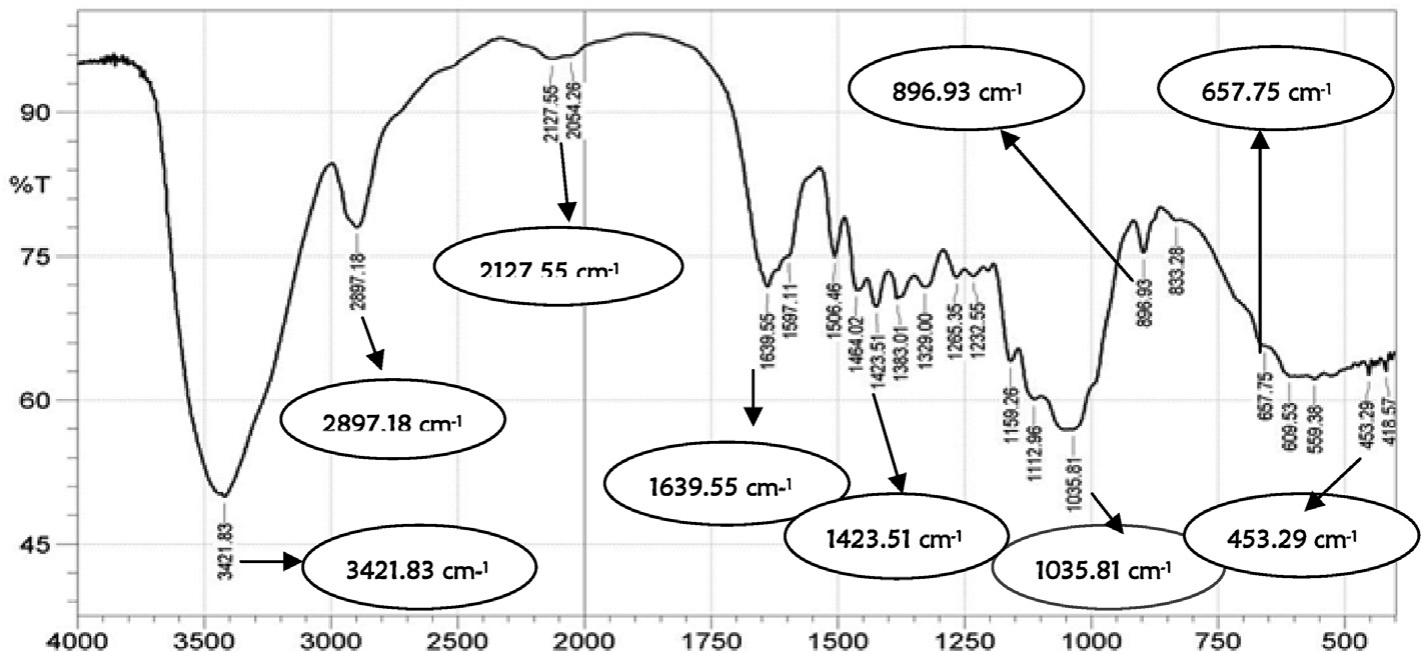
FT-IR of CNTs from Co–Fe catalyst supported on activated carbon.

The functional groups of the developed activated carbon and CNTs were determined using the FT-IR techniques in the band range of 500–4000 cm^−1^ as shown in Figures 3 and 4. The activated carbon FT-IR spectrum (Figure 3), showed that the assigned peak at 3417.98 cm^−1^ was related to the hydroxyl group (-OH) and adsorption peak at 2926.11 cm^−1^ signified the appearance of C–H stretching. The absorption bands located around 1452.45 and 1639.55 cm^−1^ were attributed to the presence of several functional groups such as C=C, C=O, and C–O. The vibration peaks at 896.93 and 1033.88 cm^−1^ depicts = C–H bending. The FT-IR results of the CNTs as shown in Figure 4 showed that the absorption band at 1035.81 cm^−1^ is attributed to the hydroxyl group (-OH), while the band at 1423.51cm^−1^ is assigned to the presence of C–C stretching. The absorption band at 2127.55 cm^−1^ was attributed to the C=C bonds, while the band at2897.18 cm^−1^ was attributed to the presence of –OH. The absorption bands at 896.93 and 1639.55, which represent the alkyl (-CH_3_) and hydroxyl (-OH), are attached to the CNTs' surface and may be responsible for adsorption of the adsorbate. The band at 3421.80 cm^−1^ is assigned to the hydroxyl functional group (–OH). The prepared CNTs revealed new bands from 1639.55 cm^−1^ and 1232.55 cm^−1^, which indicates fingerprint region of C=O, C–O and O–H groups that exist as functional groups of the CNTs. The appearance of peaks at 1597.11 and 1506.46 cm^−1^, are assigned to a conjugated hydrogen-bonded carboxyl group. The absorption bands in the closeness of 453.29 cm^−1^ and 657.75 cm^−1^ as depicted in Figure 4, could be attributed to the stretching vibrations of Co–O and Fe–O, suggesting the likely presence of cobalt oxide (CoO) and iron oxide (Fe_2_O_3_) on the carbon nanotubes structure [23].

In addition to the adsorbent active sites, the surface properties like BET surface area, pore volume and size affects the adsorption efficiency significantly during the adsorption process. The BET surface area results for the catalyst developed activated carbon and CNTs are depicted in Table 1. BET surface area results for Co–Fe/AC, activated carbon, and CNTs are 203.90, 230.24, and 372.42 m^2^/g, respectively. The high BET surface area attributed to the prepared CNTs may be as a result of the excellent support offered by the activated carbon when incorporated on the transition metals (Co–Fe) for the CNTs' development.

**Table 1:** BET analysis of the prepared AC, Co–Fe/AC and CNTs.

Sample	BET surface area (m^2^/g)	Pore volume (cm^3^/g)	Micropore volume (mg/g)	Pore size (nm)
Co–Fe/AC	203.900	0.119	0.0565	1.656
AC	230.240	0.135	0.0870	1.749
CNTs	372.420	0.186	0.0900	1.997

### 3.2 pH solution

**Figure 5:**
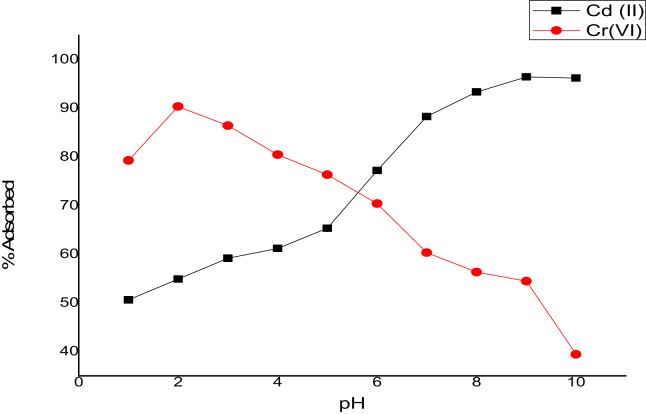
pH effect on Cd(II) and Cr(VI) adsorption by CNTs (C_o_ = 100 mg/L; Temperature = 308 K; Time = 2h; W = 0.1 g; V = 100 ml; shaking speed = 140 rpm).

A solution pH effect was conducted to establish the maximum percentage uptake of Cd(II) and Cr(VI) onto the adsorbent (CNTs) in an aqueous solution. An adjustment in the pH solution range from 1-10 was achieved with the aid 0.1 M HCl and NaOH. The effect of pH result as presented in Figure 5, showed that for Cd(II) ions, the percentage adsorbed increased from 77.24 to 96.45 % as the pH increased from 6 to 8 and remained constant as the pH increased beyond this point (pH > 8). The poor percentage uptake of Cd(II) ions at lower pH values maybe as a result of more hydrogen ion (protonation) on the adsorbent surface in an acidic medium, thereby resulting in electrostatic repulsion between the positively charged Cd(II) and positively charged adsorbent surface [22]. For Cr(VI) ions, the percentage adsorbed increased from 79.26 to 90.37 % as the pH increased from 1 to 2, and a further increase in pH (>2) resulted in percentage adsorbed decrease. The Cr(VI) ions exist as anions (HCrO_4_^-^) in an acidic medium, the adsorbent surface is protonated, and a strong electrostatic attraction occurs between the positively charged adsorbent surface and the negatively charged adsorbate, resulting in a high percentage uptake of Cr(VI). The decrease in the adsorption amount of Cr(VI), as the pH is increased, is due to the adsorbent surface being deprotonated in a basic medium, making the adsorbent surface negatively charged and resulting in electrostatic repulsion between the adsorbent and the adsorbate [24]. The solution pH of 2 and 8 was used for Cr(VI) and Cd(II) ions throughout the adsorption equilibrium process.

### 3.3 Initial metal concentration and adsorption time effect

**Figure 6:**
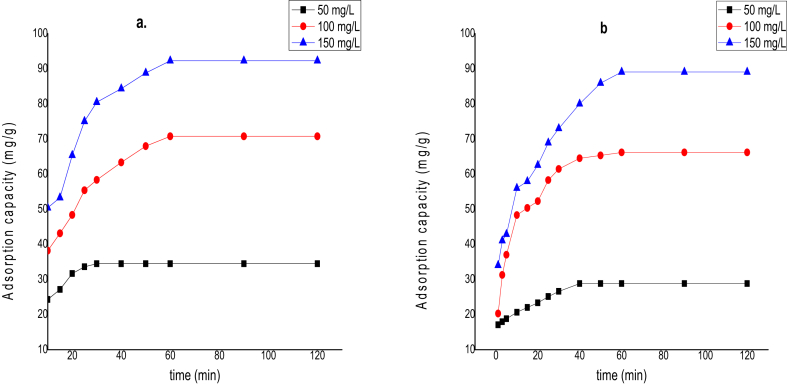
Effect of adsorption time and initial concentration on (a). Cd(II) (b). Cr(VI) adsorption onto CNTs (Temperature = 308 K; shaking speed = 140 rpm; V = 100 ml; W = 0.1 g).

The initial metal concentration (50–150 mg/L) and time (0–120 min) effect on Cd(II) and Cr(VI) ions adsorption, were studied at 308 K as plotted in Figure 6. It was noticed from the plots that the adsorbed amount increased with increasing Cd(II) and Cr(VI) ions concentration, while the adsorption extent decreased with the increasing metal ions loading [25]. At lower concentration (50 mg/L), the equilibrium position was achieved faster because there were more available binding sites on the adsorbent with fewer adsorbate competing. At 50 mg/L, the equilibrium position was attained in 30 and 40 min for Cd(II) and Cr(VI) ions. At a higher concentration (100 and 150 mg/L), it took a long time for equilibrium to be attained [26]. This is because more adsorbate molecules are competing for the few available binding sites on the adsorbent surface. At 100 and 150 mg/L, the adsorption equilibrium time was achieved in 60 min for Cd(II) and Cr(VI) ions, respectively. Nevertheless, overall contact time for the equilibrium of Cd(II) and Cr(VI) ions was measured after 2 h, to ensure that all concentration range was considered [27]. Also, the result observed that at lower concentrations of the metal ions, a greater amount of metal ions adsorbed was realized than at higher concentrations. These observations were credited to a possible proportion of vacant sites in the adsorbate molecules at those lower concentrations. Thus, they contributed immensely to the fast metal ions uptake at the adsorption process initial stages [28].

### 3.4 Adsorbent dosage

**Figure 7:**
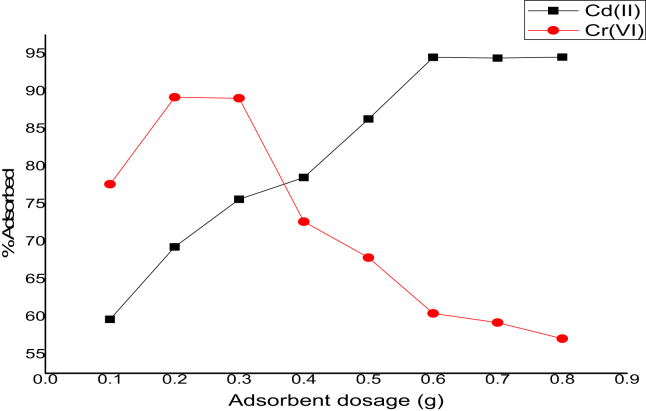
Adsorbent dosage on Cd(II) and Cr(VI) adsorption on CNTs (C_o_ = 100 mg/L; shaking speed = 140 rpm; Time = 60 min; V = 100 ml; Temperature = 308 K).

The effect of dosage at equilibrium conditions on Cd(II) and Cr(VI) ions adsorption was investigated at CNTs dosage from 0.1 to 0.8 g at initial ions concentration of 100 mg/L and adsorption time of 60 min, at 308 K, as plotted in Figure 7. It was observed that the % uptake of Cd(II) ions increased rapidly as the dosage is varied from 0.1 to 0.6 g, and dosage increase beyond this point yielded no substantial increase in the % uptake. The rapid uptake of Cd(II) as the adsorbent dosage is increased, is as a result of more vacant sites available on the adsorbent surface [29]. At little adsorbent dosage, Cd(II) ions had to contend for the inadequate pore sites on the surface of the adsorbent [30]. Rapid % removal was also observed in the case of Cr(VI) ions as the adsorbent dosage increased from 0.1 to 0.2 g, and further increase beyond this point resulted in a significant decrease in the % uptake. This observation showed that there was higher concentration number of active sites at lower adsorbent dosage. Further dosage increases beyond this point (0.2 g), led to a particle accumulation on the adsorbent active site based on the removal efficiency, thus, leading to a decrease in the % uptake of Cr(VI) ions. This finding is similar to the results obtained on the effects of pH, adsorbent dosage, time, initial concentration, and adsorption isotherm study for the removal of hexavalent chromium (Cr (VI)) from wastewater via magnetite nanoparticles [31].

### 3.5 Temperature effect

**Figure 8:**
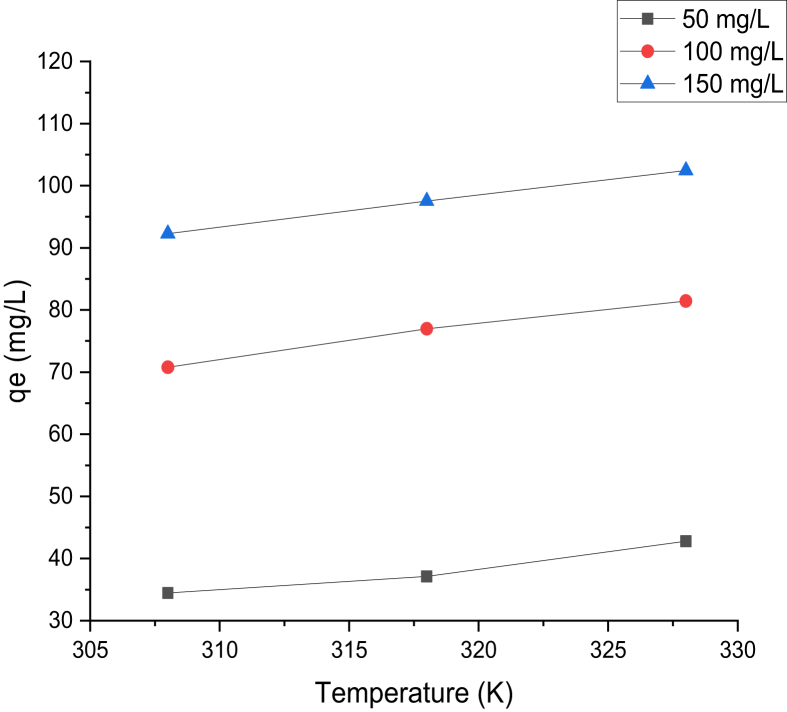
Temperature effect on the adsorption capacity of Cd(II) (Time = 60 min; pH = 8; W = 0.1 g; V = 100 ml; shaking speed = 140 rpm).

**Figure 9:**
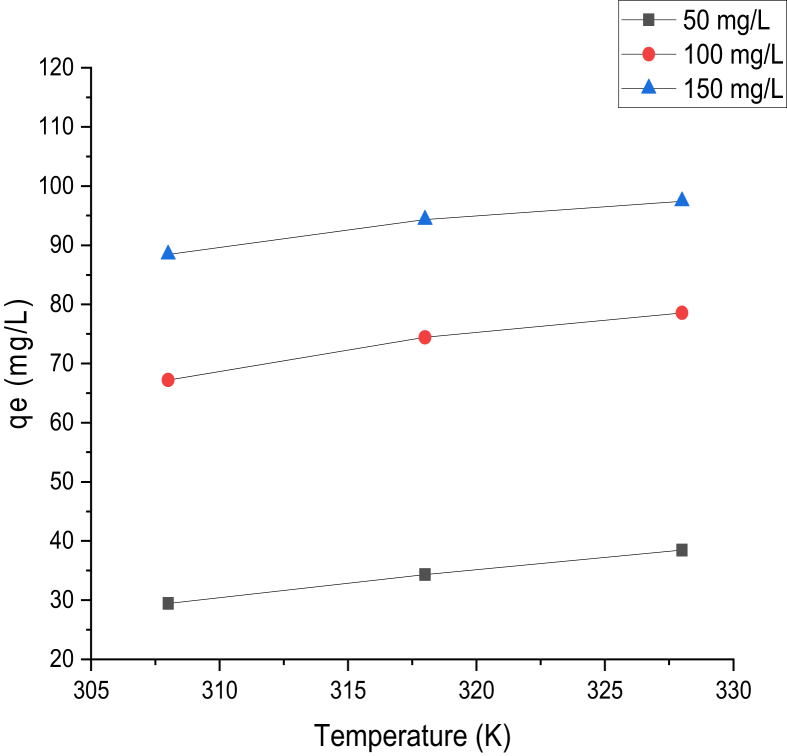
Temperature effect on the adsorption capacity of Cr(VI) (Time = 60 min; pH = 2; W = 0.1 g; V = 100 ml; shaking speed = 140 rpm).

Temperature effect on adsorption of Cd (II) and Cr(VI) ions was investigated using temperature range of 308, 318, and 328 K for 60 min, with a pH value of 2 and 8 for Cr(VI) and Cd(II) ions at different concentrations, as presented in Figures 8 and 9. The adsorption capacity of Cd(II) and Cr(VI) onto the prepared adsorbent was seen to have increased significantly with increasing temperature. This implies that, at a higher temperature, there was intraparticle diffusion increase resulting to more adsorption sites, leading to a higher adsorption capacity of Cd(II) and Cr(VI) ions, respectively. The increase in adsorption capacity of both metals, as the temperature increased, pointed to the fact that the adsorption process is endothermic.

### 3.6 Isotherms adsorption study

Langmuir model was used to express the adsorption equilibrium between the adsorbent and adsorbate system, where the adsorbate adsorption is limited to a molecular layer at, or before, a unity relative pressure was attained. The linearized form is evaluated using Eq. (3) [32].

(3) $$\frac{1}{{\text{q}}_{\text{e}}}=\frac{1}{{\text{q}}_{\text{max}}}+\frac{1}{K_{L}q_{max}C_{e}}$$

Where q_max_ is the maximum adsorption capacity of metal ions (mg/g).

Freundlich model is based on adsorption multilayer between adsorbed molecules interaction.

The linearized form is given as Eq. (4) [33].

(4) $${\text{logq}}_{\text{e}}={\text{logK}}_{\text{F}}+\frac{1}{\text{n}}{\text{logC}}_{\text{e}}$$

Where K_F_ and n are the Freundlich constant; n gives speculations about the favourability of the adsorption process and K_F_ is the metal ions adsorption capacity.

Adsorption effect on the adsorbate interaction was informed by the Temkin model.

The linearized form of the equation is given as Eq. (5) [34].

(5) $$\text{qe}=\text{B}\ln{\text{K}}_{\text{T}}+\text{B}\ln{\text{C}}_{\text{e}}$$

Where K_T_ (L/mg) is the Temkin model constant and B is the heat adsorption (kJ/mol).

(6) $$\text{B}=\frac{\text{RT}}{{\text{b}}_{\text{T}}}$$

Where $\frac{1}{b_{T}}$ indicates the adsorbent adsorption potential, R is the universal gas constant (8.314 J/mol K) and T (K) is the temperature.

The Dubinin-Radushkevich helps to determine the type of adsorption process present; whether chemisorption or physisorption. The Langmuir and Freundlich and Temkin model application do not give this type of information [35]. The Linearized form is given by Eq. (7)

(7) $$\ln\;q_{e}=\ln\;q_{s}-\text{K}{\text{ε}}^{2}$$

Where q_s_ is the saturation adsorption capacity, mg/g; K is the activity constant-coefficient- constant related to adsorption capacity; Ԑ^2^ is the Polanyi potential (kJ/mol), and E is the mean free energy of adsorption (J/mol).

The Polanyi potential is estimated using the Eq. (8)

(8) $$\text{ε}=\text{ RT In}\left(1+\frac{1}{C_{e}}\right)$$

The mean adsorption energy E in KJ/mol is estimated using Eq. (9)

(9) $$\text{E}=\frac{1}{\sqrt{2K}}$$

To further validate the fitness of these isotherm models in describing the adsorption process, error analysis methods such as chi-square (χ^2^), the sum of square error (ERRSQ/SSE), and the sum of absolute error (EABS), were applied and compared to the correlation coefficient (R^2^) obtained. The correlation coefficient (*R*^2^) only, may not validate the basis for the best adsorption model selection because it only signifies the fitness between linear forms of the isotherm equations and experimental data. Based on convergence criteria, minimization and maximization error, distribution between experimental data and predicted isotherms usually involves non-linear regression [36]. The lower the χ^2^ chi-square, ERRSQ/SSE, and EABS values for the isotherm models, studied the better fitness to the adsorption process. The error function of chi-square is calculated using Eq. (10).

(10) $$\chi^{2}\;=\sum_{i=1}^{N}\frac{{\left(q_{e,exp}-q_{e,cal}\right)}^{2}}{q_{i,cal}}$$

Sum of square error (ERRSQ/SSE) is calculated using Eq. (11).

(11) $$\text{ERRSQ/SSE}=\sum_{i=1}^{N}{\left(q_{e,cal}-q_{e,exp}\right)}^{2}$$

Sum of absolute error (EABS) equation is given by

(12) $$\text{EABS}=\sum_{i=1}^{N}\left|q_{e,exp}-q_{e,cal}\right|$$

Where N is the experimental sample number; is the experimental adsorption capacity at equilibrium (mg/g); is); $q_{i,cal}$ is the calculated adsorption capacity from the isotherm equation at equilibrium (mg/g).

**Figure 10:**
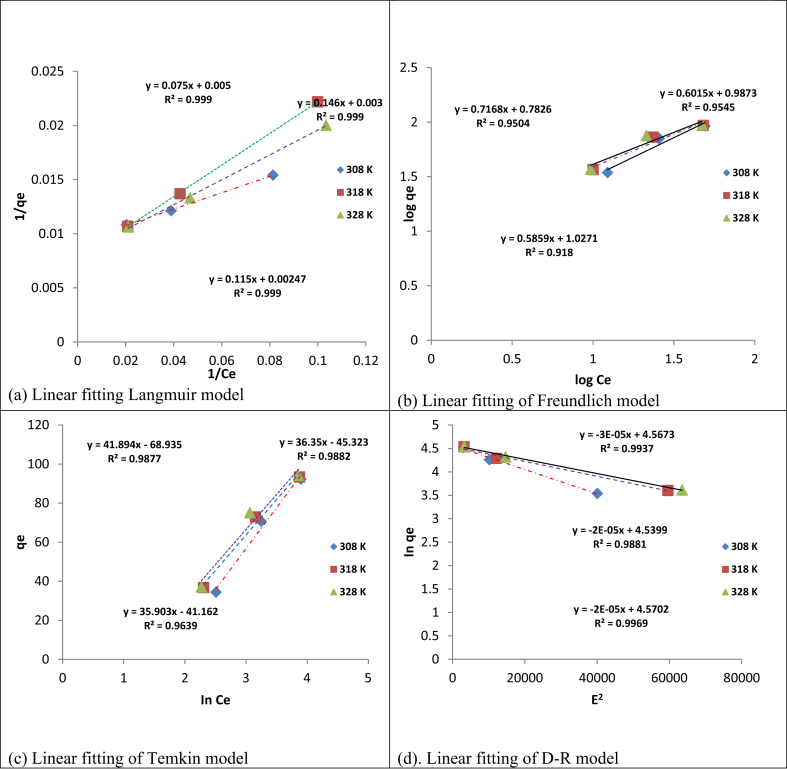
Plots for (a) Langmuir (b) Freundlich (c) Temkin and (d) D-R isotherm models for adsorption of Cd(II). (pH = 8; agitation speed = 140 rpm; contact time = 60 min; adsorbent dosage = 0.1 g).

**Figure 11:**
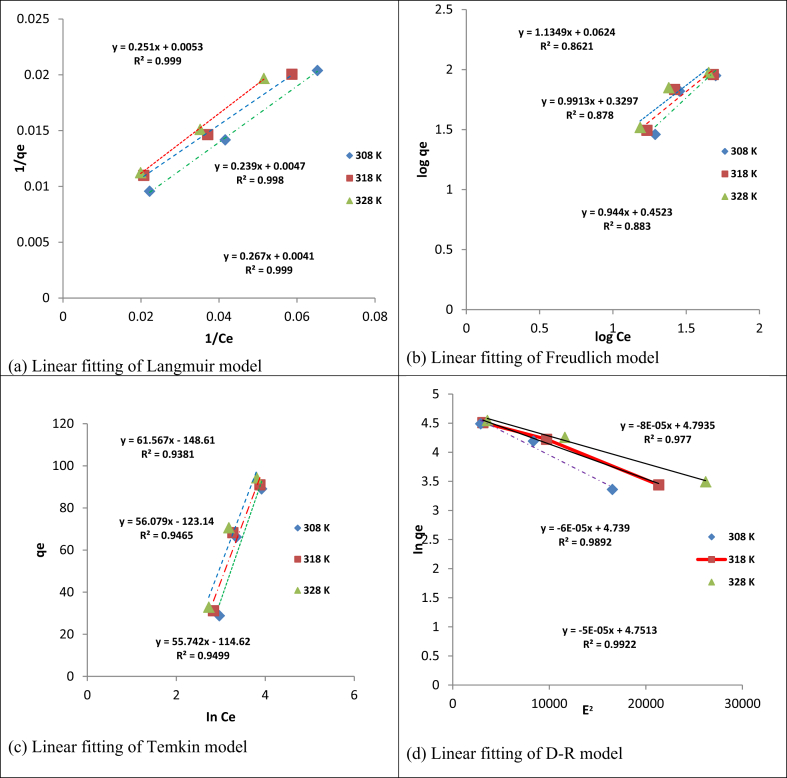
Plots for (a) Langmuir (b) Freundlich (c) Temkin and (d) D-R isotherm models for adsorption of Cr(VI). (pH = 2; agitation speed = 140 rpm; contact time = 60 min; adsorbent dosage = 0.1 g).

Adsorption isotherm model parameters and the error analysis results are presented in Table 2 and Figures 10 and 11. The result clearly showed that Langmuir isotherm amongst the other isotherms studied, best described the adsorption of Cd(II) and Cr(VI) ions with higher correlationcoefficientR^2^ (>0.99), followed by Dubinin-Radushkevich, Temkin, and Freundlich model, respectively. In addition, this assertion was further justified with lower values of chi-square (χ^2^), the sum of square error (ERRSQ/SSE), and the sum of absolute error (EABS), obtained when compared with the order models indicating the closeness of the model experimental results to the calculated results obtained [36, 37, 38]. This finding is similar to the results obtained on the sorption of cadmium and hexavalent chromium from electroplating wastewater [23] and non-linear prediction of kinetic and equilibrium data for the adsorption of hexavalent chromium by carbon materials [39]. According to the Langmuir model, the process adsorption followed monolayer and homogeneous mechanism of adsorption with a maximum adsorption capacity of 404.858 and 243.902 mg/g for Cd(II) and Cr(VI) ions, respectively. The high uptake capacity of Cd(II) over Cr(VI) ions is as a result of their ionic properties (see Table 3). The high adsorption capacity of Cd(II) when compared with Cr(VI), showed that Cd (II) ions are more accessible to the adsorbent pore as informed by its smallest hydrated radii value [40]. Furthermore, the highest standard reduction potential value of Cd(II) ions, showed that it exhibited stronger ionic interaction with the electron-rich adsorbent surface [41]. Finally, Cd(II) ion has the most electronegativity value, implying that it is more attracted to the adsorbent surface [42, 43]. The comparison of the prepared adsorbent with other natural and synthetic adsorbents is presented in Table 4. The mean adsorption energy values of the D-R model in Table 2 for both adsorbed metals were seen to be less than 8 kJ/mol (E < 8 kJ/mol). This is an indication that there was a possibility of physical interaction of Cd(II) and Cr(VI) on the adsorbent surface.

**Table 2:** Langmuir Freundlich, Temkin and Dubinin–Radushkevich isotherm models parameters for Cd(II) and Cr(VI) uptake.

Isotherms	Parameters	Cd(II)			Cr(VI)		
		35 °C	45 °C	55 °C	35 °C	45 °C	55 °C
Langmuir	q_m_ (mg/g)	200.000	333.333	404.858	188.679	212.766	243.902
	K_L_ (L/mg)	0.0667	0.0205	0.0215	0.0211	0.0197	0.0154
	R^2^	0.999	0.999	0.999	0.999	0.998	0.999
	χ^2^	0.119	0.0489	0.0348	0.0393	0.125	0.186
	ERRSQ/SSE	8.179	2.298	1.900	1.489	10.636	13.759
	EABS	4.300	1.720	0.900	2.030	3.980	2.810
Freundlich	K_F_	6.0534	9.705	10.641	1.153	2.133	2.831
	1/n	0.716	0.601	0.585	1.134	0.991	0.944
	R^2^	0.950	0.954	0.918	0.862	0.878	0.883
	χ^2^	21.237	24.142	52.021	55.921	28.670	50.135
	ERRSQ/SSE	80.951	87.688	164.816	821.654	640.251	863.3122
	EABS	42.410	33.410	60.380	144.910	129.150	150.580
Temkin	K_T_ (L/mg)	0.190	0.290	0.310	0.0890	0.110	0.130
	B (kJ/mol)	41.890	36.350	35.900	61.560	56.070	55.750
	R^2^	0.987	0.988	0.963	0.938	0.946	0.949
	χ^2^	1.263	1.584	3.971	2.231	1.478	1.709
	ERRSQ/SSE	49.206	62.416	123.028	114.840	34.932	43.678
	EABS	1.940	7.830	16.760	5.720	6.040	5.370
Dubinin-Radushkevich (D-R)	q_s_ (mg/g)	96.255	93.597	96.544	120.663	114.320	115.700
	K (mol^2^/J^2^)10^−5^	3.000	2.000	2.000	8.000	6.000	5.000
	E (kJ/mol)	0.129	0.158	0.112	0.0791	0.0913	0.992
	R^2^	0.993	0.988	0.996	0.977	0.989	0.992
	χ^2^	0.342	0.307	0.965	1.114	0.799	0.762
	ERRSQ/SSE	22.185	19.772	63.196	75.960	98.646	98.318
	EABS	1.0700	2.960	2.680	1.020	1.950	2.700

**Table 3:** Ionic property of Cd(II) and Cr(VI) ions.

Property	Cd(II)	Cr(VI)
Molecular weight	769.52	294.18
Atomic weight	112.41	51.01
Electronic Configuration	[Kr]4^d^5s^2^	[Ar]3d^5^4s^1^
Hydrated radii (A^o^)	4.260	4.61
Ionic radii (A^o^)	0.95	0.52
Standard Reduction Potential (V)	Cr^6+^ + 3e^−^→Cr^3+^ (1.1)	Cd^2+^ + 2e^−^→Cd (-0.403)
	Cr^3+^→Cr (-0.74)	
Coordination number	6 & 4	6 & 4
Electronegativity	1.69	1.66

Source: [44].

**Table 4:** Comparison of Cd(II) and Cr(VI) adsorption capacity with various adsorbent.

Adsorbent	Metal uptake capacity (mg/g)		References
	Cd(II)	Cr(VI)	
Phosphogysum	131.5		[45]
Olive cake	64		[46]
Coconut coir pith		76.3	[47]
Dolochar	1.9	2.1	[48]
Sulfurized activated carbon	104.17		[49]
Sulfonated Graphene Nanosheets	58		[50]
Chitosan/Sulfydryl-functionalized	167		[51]
Boron waste	105		[24]
Modified activated carbon		49.9	[52]
Multiwalled carbon nanotubes			[53]
MWCNTs from Co–Fe/AC	404.858	243.902	Present study

### 3.7 Adsorption kinetics

Adsorption kinetics of the metal ions was studied using the following pseudo-first-order, second-order Elovich and intraparticle diffusion kinetic models to examine and explain the adsorption mechanisms between the adsorbate and adsorbent. The first-order, second-order elovich and intraparticle diffusion kinetic models were evaluated using Eqs. (13), (14), (15), and (16) [54].

(13) $$\log\left({\text{q}}_{\text{e}-}{\text{q}}_{\text{t}}\right)=\log{\text{q}}_{\text{e}-}\frac{{\text{k}}_{1}\text{t}}{2.303}$$

(14) $$\frac{\text{t}}{{\text{q}}_{\text{t}}}=\frac{\text{t}}{{\text{q}}_{\text{e}}}+\frac{1}{{\text{k}}_{2}{\text{q}}_{\text{e}}^{2}}$$

(15) $$q_{t}=\frac{1}{\beta }\ln\left(\alpha \beta \right)+\frac{1}{\beta }\ln\;t$$

(16) $$q_{t}=k_{p}t^{0.5}+\;C$$

Where k_1_ and k_2_are the pseudo-first order and second-order rate constant in min^−1^ and g/mgmin, respectively; C is the plotted intercept; k_p_ is the rate constant of the intraparticle; and α and β is Elovich constant which are obtained from plots of q_t_ against lnt.

**Figure 12:**
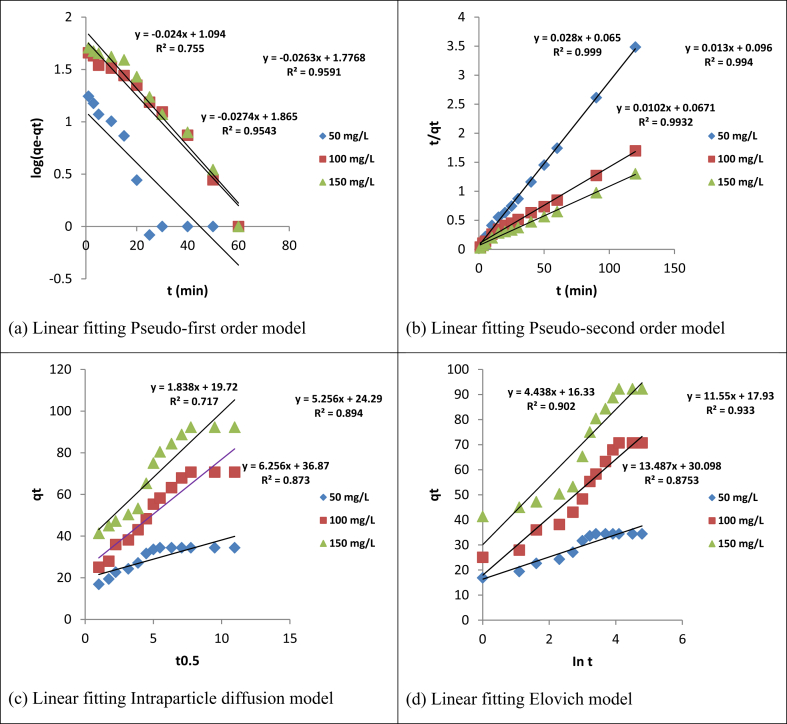
Plots for (a) Pseudo-first order (b) Pseudo-second order (c) Intraparticle diffusion and (d) Elovich kinetics models for Cd(II) uptake at 308 K.

**Figure 13:**
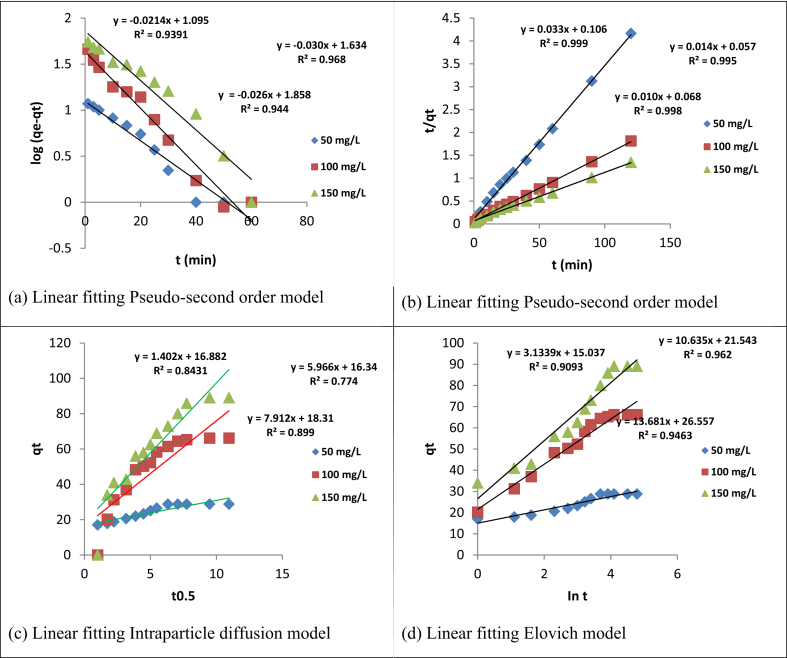
Plots for (a) Pseudo-first order (b) Pseudo-second order (c) Intraparticle diffusion and (d) Elovich kinetic models for Cr(VI) uptake at 308 K.

To investigate the kinetic models for the adsorption of Cd(II) and Cr(VI), four kinetic models namely; pseudo-first-order, pseudo-second-order, Elovich, and intraparticle diffusion were tested and their regression coefficients, error functions, and information criteria are presented in Table 5 and their plots in Figures 12 and 13. The results showed that pseudo-second-order model, when compared with the other models, described best the adsorption of Cd(II) and Cr(VI) ions judging by their regression coefficient (R^2^)and Adj. R^2^values (>0.99) with possible chemical interaction; then followed by pseudo-first-order, elovich, and intraparticle diffusion models respectively, for the studied range of concentration [55]. The suitability of pseudo-second-order kinetic model to fitting the adsorption data was further attested to by the maximum log-likelihood values and smaller ERRSQ/SSE, χ^2^, AIC, BIC and HQIC values. It was further observed (Table 5) that positive values of log-likelihood resulted in negative criterion values (AIC, BIC and HQIC), while negative criterion values resulted in positive log-likelihood values, respectively. Close observation revealed that the pseudo-second-order equilibrium experimental adsorption capacity (q_e_ exp) was closer to the calculated (q_e_cal) when compared with the pseudo-first-order. This finding was further supported by a significant decrease in the pseudo-second-order adsorption rate constant (k_2_); which attests to the fact that Cd(II) and Cr(VI) adsorption attain equilibrium faster at lower initial concentration [56]. The intraparticle diffusion model plots for adsorption of both metal ions were seen to have two different phases. This includes; the steep sides of the plots, which indicate the boundary layer and the linear portion, which represent the intraparticle diffusion [28]. It was further observed from the plots that the steep side, which is the boundary layer, is higher than the intraparticle which is the linear portion. The plots of q_t_ versus t^0.5^ for both metals adsorbed are not linear graphs thereby suggesting that the rate-determining steps are not intraparticle diffusion. Therefore, the adsorption kinetic study indicates that Cd(II) and Cr(VI) adsorption may be governed simultaneously by pseudo-second-order kinetics, intraparticle diffusion, and interfacial diffusion. Furthermore, both metals ions are transported from the solution bulk to the adsorbent external surface and are later transferred into the active sites of interlayer space [57].

**Table 5:** Kinetic models constants with the correction coefficients for Cd(II) and Cr(VI) uptake at 308 K.

Kinetic model	Parameters	Cd(II)			Cr(VI)		
	C_o_ (mg/L)	50	100	150	50	100	150
Pseudo-first order	q_e_ exp (mg/g)	34.450	70.760	92.280	28.820	66.190	89.080
	q_e_cal (mg/g)	12.417	59.704	73.282	12.445	43.052	72.111
	k_1_ (min^−1^)	0.0240	0.0260	0.270	0.021	0.0300	0.0260
	R^2^	0.755	0.959	0.954	0.939	0.968	0.944
	Adj. R^2^	0.727	0.955	0.949	0.932	0.964	0.937
	SSE	0.751	0.959	0.139	0.115	0.122	0.167
	SIC	6.488	-14.204	-12.054	-14.143	-13.491	-10.060
	AIC	5.692	-15.000	-12.850	-14.939	-14.287	-10.856
	HQC	5.191	-15.502	-13.351	-15.440	-14.789	-11.358
	Log-Likelihood	-0.846	9.500	-8.424	9.469	9.143	7.428
	χ^*2*^	2.394	0.506	1.476	6.004	1.015	0.371
Pseudo- second order	q_e_cal (mg/g)	35.714	76.923	100.000	30.303	71.429	100.000
	k_2_ (g/mg min)	0.0121	0.00176	0.00149	0.0103	0.00344	0.00147
	R^2^	0.999	0.994	0.993	0.999	0.995	0.998
	Adj. R^2^	0.999	0.993	0.993	0.999	0.995	0.998
	SSE	0.0151	0.0167	0.0111	0.00370	0.00870	0.0329
	SIC	-45.851	-44.535	-49.791	-64.112	-53.001	-35.700
	AIC	-46.981	-45.664	-50.921	-65.242	-54.132	-36.830
	HQC	-47.213	-45.897	-51.153	-65.474	-54.364	-37.0620
	Log-Likelihood	25.490	24.832	27.460	34.621	29.0660	20.415
	χ^*2*^	2.423	0.164	1.215	0.676	0.258	0.832
Elovich	β	0.225	0.0866	0.0742	0.319	0.0941	0.0731
	α	175.196	54.534	125.611	379.890	80.705	52.163
	R^2^	0.902	0.933	0.875	0.909	0.962	0.946
	Adj. R^2^	0.893	0.927	0.864	0.901	0.959	0.941
	SSE	49.803	223.379	602.542	22.780	103.907	247.0260
	SIC	59.483	78.993	91.893	49.314	69.0430	80.301
	AIC	58.353	77.863	90.763	48.184	67.914	79.171
	HQC	58.121	77.631	90.531	47.952	67.681	78.939
	Log-Likelihood	-27.176	-36.932	-43.382	-22.0920	-31.957	-37.585
	χ^*2*^	0.907	0.145	0.646	3.218	1.032	1.345
Intraparticle diffusion	k_p_ (mg/g min^0.5^)	1.838	5.256	6.356	1.402	5.996	7.912
	C	19.720	24.290	36.870	16.880	16.340	18.310
	R^2^	0.717	0.894	0.873	0.843	0.774	0.899
	Adj. R^2^	0.691	0.884	0.861	0.829	0.753	0.890
	SSE	143.926	353.357	615.805	39.384	618.139	463.377
	SIC	73.279	84.955	92.176	56.431	92.225	88.479
	AIC	72.149	83.825	91.046	55.302	91.095	87.349
	HQC	71.917	83.593	90.814	55.069	90.863	87.117
	Log-Likelihood	-34.0740	-39.912	-43.523	-25.651	-43.547	-41.675
	χ^*2*^	0.878	0.653	0.523	0.127	4.445	1.005

### 3.8 Adsorption thermodynamics

The parameters ΔG^o^, ΔH^o^ and ΔS^o^ of the adsorption thermodynamic tested at different temperature is given by Eq. (17) [21].

(17) $$\text{log}\frac{{\text{q}}_{\text{e}}}{{\text{C}}_{\text{e}}}=-\frac{\Delta {\text{H}}^{\text{O}}}{2.303\text{R}}\left(\frac{1}{\text{T}}\right)+\frac{\Delta {\text{S}}^{\text{O}}}{2.303\text{R}}$$

The free Gibbs energy ΔG^o^ is evaluated using Eq. (18)

(18) ΔG^o^ = ΔH^o^ -TΔS^o^

The adsorption thermodynamics parameters for Cd(II) and Cr(VI) ions adsorption onto MWCNTs is summarized in Table 4. The entropy (ΔS^o^) and enthalpy (ΔH^o^) results were calculated from the intercept and slope of the linear graph of $\text{log}\frac{{\text{q}}_{\text{e}}}{{\text{C}}_{\text{e}}}$ against $\frac{1}{\text{T}}$. The negative ΔG^o^ and positive ΔH^o^ values indicated the spontaneity and endothermic nature (temperature increased resulted to increase metal ions uptake capacity) of the adsorption mechanism for both metal ions as depicted in Table 6. The positive values of ΔS^o^ showed an affinity of the adsorbent towards the adsorbate and there was also greater randomness at the adsorbent-adsorbate interface [58, 59].

**Table 6:** Thermodynamic adsorption values for Cd(II) and Cr(VI) ions uptake.

Adsorbate	q_e_ (mg/g)	ΔH^o^ (J/mol)	ΔS^o^ (J/K mol)	ΔG^o^ (kJ/mol)		
				308 K	318 K	328 K
Cd(II)	34.450	1.245	2.202	-0.679	-0.701	-0.724
	70.760			-0.833	-0.860	-0.881
	92.280			-0.857	-0.884	-0.912
Cr(VI)	28.820	1.781	0.670	-0.208	-0.215	-0.222
	66.190			-0.0136	-0.0140	-0.0144
	89.080			-0.0493	-0.0507	-0.0523

The adsorption activation energy (E_a_) was calculated from the Arrhenius equation as given in Eq. (19)

(19) $$\ln\;k_{2}=-\frac{E_{a}}{R}\left(\frac{1}{T}\right)+\ln\;A$$

where R is the gas rate constant (8.314 J/mol K) and E_a_ is the activation energy (kJ/mol) and A is the Arrhenius constant. The E_a_ value between 5- 40 kJ/mol connotes physiosorption while a value between 40-800 kJ/mol implies chemisorption [60, 61].

**Figure 14:**
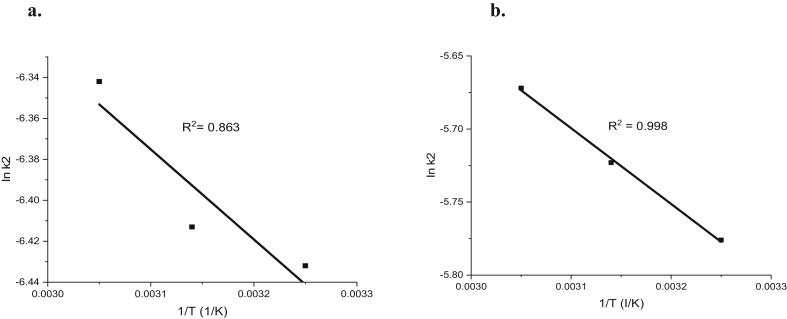
Arrhenius plots of Ink_2_ against 1/T for (a) Cd(II) and (b) Cr(VI) adsorption on the surface of Multiwall carbon nanotubes.

The experimental values of contact time effect on the adsorption capacity of Cd(II) and Cr(VI) at 308, 318, and 328 K were used. Assuming pseudo-second-order kinetics, the slope (k_2_) values were obtained at 308, 318, and 328 K from the plots of t/qt against t. The activation energy (E_a_) values were calculated from Arrhenius plots of ln k_2_ versus 1/T in Figure 14. The E_a_ values of 51.131 and 45.0683 kJ/mol were obtained for Cd(II) and Cr(VI) respectively as presented in Table 7. The E_a_ values obtained revealed that the adsorption of Cd(II) and Cr(VI) by MWCNTs was more dominated by chemical adsorption rather physical adsorption.

**Table 7:** Adsorption activation energy values.

Adsorbent	E_a_ (kJ/mol)	A	R^2^
Cd(II)	51.131	0.00602	0.863
Cr(VI)	45.0683	0.0167	0.998

## 4 Conclusions

Activated carbon, derived from castor seed waste, was supported on the cobalt-ferrite catalyst for developing multiwall carbon nanotubes (MWCNTs) and was then used as an adsorbent for the effective removal of Cd(II) and Cr (VI) for an aqueous solution. The textural properties, functional groups, and morphological structures of the prepared activated carbon and MWCNTs adsorbent, were determined by using BET, FT-IR, and SEM. The adsorption isotherm model was best described by Langmuir isotherm with maximum adsorption capacities of 243.902 and 404.858 mg/g, at pH 2 and 8 for Cr(VI) and Cd(II), respectively. The kinetic model was applied to the adsorption equilibrium data in other to predict the adsorption mechanism of the adsorbent. The result showed that adsorption followed the pseudo-second-order kinetic model. The adsorption thermodynamic parameters showed that the adsorption of Cd(II) and Cr(VI) progressed spontaneously with an endothermic nature, and exhibited randomness increased during the adsorption process with increasing temperature. The adsorption mechanism of Cd(II) and Cr(VI), revealed the combination of dominated chemical adsorption with subordinate physical adsorption. In addition, the development of MWCNTS prepared from activated carbon as an adsorbent could be utilized successfully for the treatment of water containing Cd(II) and Cr(VI) ions because of its cost-effectiveness, reusability, high surface area, and high adsorption capacity.

## Declarations

### Author contribution statement

K. S. Obayomi: Conceived and designed the experiments; Performed the experiments; Analyzed and interpreted the data; Contributed reagents, materials, analysis tools or data; Wrote the paper.

J. O Bello: Conceived and designed the experiments.

M.D. Yahya: Analyzed and interpreted the data; Wrote the paper.

E., Chukwunedum & J. B., Adeoye: Performed the experiments; Contributed reagents, materials, analysis tools or data.

### Funding statement

This research did not receive any specific grant from funding agencies in the public, commercial, or not-for-profit sectors.

### Competing interest statement

The authors declare no conflict of interest.

### Additional information

No additional information is available for this paper.
